# Clinical characteristics, semiology, and comorbidity patterns in non-epileptic seizures (NES): a retrospective comparative study

**DOI:** 10.3389/fpsyg.2026.1982723

**Published:** 2026-10-22

**Authors:** Paola Caruso, Magda Quagliotto, Yvonne Radin, Stefania Pavan, Paolo Manganotti, Alberto Benussi

**Affiliations:** 1 Neurology Unit, Department of Medical, Surgical and Health Sciences, University of Trieste, Trieste, Italy; 2 S.C.U. Obstetrics and Gynaecology Clinic, Institute for Maternal and Child Health, IRCCS “Burlo Garofolo, ” Trieste, Italy

**Keywords:** EEG, epilepsy, functional neurological disorder, neurological disorder, non-epileptic seizures

## Abstract

**Objectives:**

Non-epileptic seizures (NES) are paroxysmal events resembling epileptic seizures but not explained by ictal activity. Differential diagnosis may be challenging, particularly because NES and epilepsy can coexist. We aimed to characterize the demographic, clinical, semiological, psychiatric, and instrumental profile of patients with NES, compare patients with NES only with patients with NES epilepsy-only, and assess the robustness of the main associations through sensitivity analyses.

**Methods:**

We conducted a retrospective observational comparative study using a clinical database from our Neurology Unit (2022–2025) including 38 patients with NES and 23 patients with epilepsy without NES. A total of 18 NES patients had comorbid epilepsy; therefore, the primary comparison included mutually exclusive groups: NES-only (*n* = 20) and epilepsy-only (*n* = 23). All NES patients were retained for descriptive analyses. Between-group comparisons used exact or non-parametric tests with Benjamini–Hochberg false-discovery-rate (BH-FDR) correction. Sensitivity analyses restricted NES-only patients to those with database-recorded video-EEG confirmation and used Firth penalized logistic regression adjusted for age and sex.

**Results:**

Among 38 patients with NES, 94.7% had documented psychological/psychiatric comorbidity, mainly anxiety (50.0%) and depression (28.9%). Positive motor phenomena were documented in 73.7%, neurological examination inconsistency/incongruity in 85.7% of evaluable patients, and video-EEG confirmation in 73.7%. Psychological/psychiatric comorbidity was significantly more frequent in NES-only than epilepsy-only patients (90.0% vs. 13.0%; exact OR 50.40, 95% CI 7.29–670.28; BH-FDR *p* < 0.001), as was anxiety (45.0% vs. 4.3%; BH-FDR *p* = 0.014). Broad seizure-semiology domains were not significantly different after multiplicity correction. Among imaged patients, abnormal MRI findings were more frequent in epilepsy-only patients (12/18 vs. 0/10; BH-FDR *p* = 0.006), although MRI ascertainment differed between groups. The psychiatric-comorbidity association persisted in sensitivity analyses.

**Conclusion:**

NES showed substantial clinical heterogeneity and frequently coexisted with epilepsy. Psychological/psychiatric comorbidity was markedly more frequent in NES-only than epilepsy-only patients and remained robust in sensitivity analyses, although differential ascertainment limits interpretation. Broad seizure manifestations alone did not distinguish the groups after multiplicity correction. Instrumental findings should be considered descriptive rather than NES biomarkers. These findings support explicit stratification for comorbid epilepsy and standardized prospective assessment of psychiatric and semiological features.

## Introduction

Psychogenic non-epileptic seizures (NES), for which the International League Against Epilepsy (ILAE) has recently proposed the term functional/dissociative seizures (FDS), are paroxysmal events involving alterations in awareness, perception, behavior, motor function, or autonomic experience that can resemble epileptic seizures but are not themselves explained by epileptic ictal activity (Asadi-Pooya, 2023; Hingray et al., 2025; Reuber et al., 2002). NES represent a major diagnostic and therapeutic challenge because of their phenotypic overlap with epilepsy and the frequent delay in establishing the correct diagnosis. A substantial proportion of patients referred to epilepsy monitoring units for apparently drug-resistant events are ultimately diagnosed with NES (Anzellotti et al., 2020; Kanemoto et al., 2017; LaFrance et al., 2013). Delayed recognition may lead to repeated emergency department visits, hospitalizations, investigations, and unnecessary treatment. Careful clinical assessment and video-EEG when appropriate are therefore important components of diagnostic evaluation, while recognizing that not every patient has access to event capture on video-EEG.

Non-epileptic seizures are frequently associated with psychological and psychiatric comorbidities, including depression, anxiety disorders, post-traumatic stress symptoms, personality disorders, and other psychiatric conditions (Lanzillotti et al., 2021). Reported prevalence varies substantially according to population and ascertainment method, and psychiatric history alone is not diagnostic of NES (Brown and Reuber, 2016; Gasparini et al., 2019; Walsh et al., 2018). Epilepsy may coexist with NES in a clinically important subset of patients, resulting in a dual diagnosis; systematic-review evidence indicates that this coexistence is not uncommon in specialized clinical settings (Kutlubaev et al., 2018). Consequently, neither psychiatric comorbidity nor a previous diagnosis of epilepsy excludes NES.

Diagnostic uncertainty may also lead to inappropriate or prolonged antiseizure medication (ASM) exposure when non-epileptic events are interpreted as drug-resistant epilepsy. ASMs do not treat NES events themselves, although they may remain indicated when epilepsy coexists; furthermore, they sometimes have dual indications and may be continued for psychiatric co-morbidity reasons (e.g., valproic acid and lamotrigine for bipolar disorder). Failure to distinguish the two event types may therefore expose patients to avoidable medication burden and acute interventions while delaying access to appropriate explanation, psychological treatment, and multidisciplinary care (Anzellotti et al., 2020; Jungilligens et al., 2021; Mostacci et al., 2011; Viarasilpa et al., 2019; von Elm et al., 2007; Walsh et al., 2018). Video-EEG remains the gold standard for the diagnosis of NES, as it allows the correlation of the observed event with the absence of an epileptic ictal EEG correlate; however, when video-EEG monitoring is not feasible, smartphone-recorded videos of habitual events may provide useful additional information on seizure semiology and support the diagnostic assessment when reviewed by an experienced epileptologist (Whitehead et al., 2017).

This study aimed to (1) describe the demographic, clinical, semiological, psychiatric, and instrumental characteristics of a NES cohort; (2) compare mutually exclusive NES-only and epilepsy-only groups using harmonized variables; (3) explore differences between NES patients with and without comorbid epilepsy; and (4) assess the robustness of the principal between-group association in sensitivity analyses accounting for database-recorded video-EEG confirmation and for age and sex.

## Materials and methods

### Study design and population

#### Methods

We conducted a retrospective observational comparative study based on a clinical database collected at the Neurology Unit in Trieste, North-Eastern Italy, from 2022 to 2025. The analytic dataset comprised 38 patients with NES and 23 comparison patients with epilepsy and no NES. Within the NES cohort, 18/38 had comorbid epilepsy and 20/38 did not. Demographic and clinical variables available in both databases were harmonized before comparative analysis. Occupation and educational level were not analyzed because of extensive missingness.

Patients with NES had been evaluated either after presentation to the Emergency Department for suspected seizure-like events or following outpatient neurological assessment. The NES diagnosis was established by a neurologist on the basis of the clinical history and event semiology, supported by EEG/video-EEG when available, particularly, the diagnosis of NES was supported for all patients by the performance of at least one event-related EEG, which made it possible to rule out the presence of epileptiform activity as the aetiological cause of the symptoms observed clinically and/or reported by the patient. Furthermore, in 28 out of 38 patients (73.7%), it was possible to gain a more detailed understanding of the clinical picture by performing a video-EEG lasting at least 24 h, which allowed the video recording of the symptoms to be correlated with the EEG recording as evidence of epileptiform activity. Unfortunately, although video-EEG is the standard procedure that should be carried out in all cases, this was not possible in 10 out of 38 cases (for example, due to the patient’s unwillingness to be admitted to hospital for a few days, etc.); in these cases, the clinician therefore relied on the EEG recording. Brain MRI was used to evaluate alternative or coexisting neurological pathology rather than as a diagnostic test for NES. The epilepsy comparison group comprised patients with epilepsy managed in the Epilepsy Clinic and confirmed to have no NES.

For the NES group, all patients aged 18–50 years who presented to the Emergency Department or underwent an urgent neurological evaluation for suspected epileptic seizures were considered eligible. Patients with previous Emergency Department visits for suspected functional neurological disorder and those with other acute medical conditions were excluded. The control group consisted of age-matched patients with a history of epilepsy for at least 5 years, with no other significant medical or neurological comorbidities. The research was conducted in line with the principles of the Declaration of Helsinki and was approved by the Local Ethics Committee (University of Trieste).

### Clinical evaluation

Non-epileptic seizures semiology was coded from the original clinical event descriptions using operational domains defined before the final coding pass. Domains were not mutually exclusive and included positive motor phenomena; negative motor phenomena; impaired responsiveness/contact; preserved awareness/responsiveness; falls/collapse; speech/language disturbance; sensory/perceptual symptoms; dissociative/derealization phenomena; autonomic/visceral symptoms; and prolonged events (operationally defined as ≥10 min). Negative motor phenomena were counted only when weakness, paresis, motor arrest, or inability to move was explicitly part of the paroxysmal event; interictal weakness and neurological-examination findings were not counted as ictal semiology. Positive functional ictal features were analyzed separately and comprised explicitly documented ictal variability/fluctuation/incongruity, distractibility/modifiability, asynchronous/asymmetric motor activity, ictal eye closure or opposition to passive eye opening, and rapid recovery or explicit absence of postictal confusion. An explicit temporal or modulatory association with stress or emotional context was treated as a contextual feature rather than as a positive diagnostic sign. Lack of documentation was coded as not documented and was not interpreted as proof of clinical absence. Because epilepsy-control event descriptions were generally less detailed, only broad domains with sufficiently comparable ascertainment were used in between-group semiology comparisons.

#### Statistical analysis

The primary diagnostic-group comparison therefore used mutually exclusive groups: NES without comorbid epilepsy (NES-only, *n* = 20) versus epilepsy without NES (epilepsy-only, *n* = 23). All 38 NES patients were retained for descriptive phenotyping. A secondary within-NES comparison evaluated NES-only (*n* = 20) versus NES with comorbid epilepsy (*n* = 18), and an exploratory three-group analysis considered NES-only, NES + epilepsy, and epilepsy-only separately.

Continuous variables are summarized as median [interquartile range] because of the modest sample size and distributional heterogeneity and were compared with the two-sided Mann-Whitney U test; rank-biserial correlation is reported as effect size. Binary variables are reported as *n*/*N* (%) and compared with two-sided Fisher exact tests, with conditional maximum-likelihood odds ratios and exact 95% confidence intervals. Given the exploratory nature of the study, Benjamini-Hochberg false-discovery-rate (BH-FDR) adjustment was applied across the 26 testable between-group comparisons in Table 1; rows with zero events in both groups were retained descriptively but excluded from inferential testing. For the secondary NES-only versus NES + epilepsy analysis, BH-FDR adjustment was applied once across all 12 comparisons in Table 3. Three-group categorical comparisons used an exact Fisher-Freeman-Halton procedure with Cramer’s V as effect size. Statistical significance was interpreted together with effect size, confidence intervals, denominator, ascertainment comparability, and clinical context; statistical significance was not equated with causality, diagnostic specificity, or equivalence when a comparison was non-significant.

**TABLE 1:** Clinical characteristics and exploratory comparisons between non-epileptic seizures (NES)-only and epilepsy-only groups.

Variable	NES-only (*n* = 20)	Epilepsy-only (*n* = 23)	Effect estimate	*P*-value	BH-FDR *p*
Demographic and clinical context					
Age at first neurological visit, years	31.0 [24.8–49.8]	41.0 [29.0–60.5]	Rank-biserial *r* = −0.28	0.122	0.385
Female sex	17/20 (85.0%)	15/23 (65.2%)	OR 2.95 (95% CI 0.57–20.42)	0.175	0.414
Recorded neurological comorbidity	7/20 (35.0%)	9/23 (39.1%)	OR 0.84 (95% CI 0.20–3.43)	1.000	1.000
Family history of epilepsy	1/20 (5.0%)	3/23 (13.0%)	OR 0.36 (95% CI 0.01–4.92)	0.610	0.835
Current/previous substance use	1/20 (5.0%)	2/23 (8.7%)	OR 0.56 (95% CI 0.01–11.57)	1.000	1.000
Documented psychological/psychiatric comorbidity					
Any documented psychological/psychiatric comorbidity	18/20 (90.0%)	3/23 (13.0%)	OR 50.40 (95% CI 7.29–670.28)	**<0.001**	<0.001
Anxiety	9/20 (45.0%)	1/23 (4.3%)	OR 16.82 (95% CI 1.93–823.67)	**0.003**	0.014
Depression	7/20 (35.0%)	3/23 (13.0%)	OR 3.48 (95% CI 0.65–24.70)	0.148	0.385
Panic attacks	3/20 (15.0%)	1/23 (4.3%)	OR 3.77 (95% CI 0.27–212.88)	0.323	0.601
Bipolar disorder	1/20 (5.0%)	0/23 (0.0%)	OR ∞ (95% CI 0.03–∞)	0.465	0.672
Other psychological/psychiatric comorbidity	1/20 (5.0%)	1/23 (4.3%)	OR 1.15 (95% CI 0.01–94.97)	1.000	1.000
Psychosis	2/20 (10.0%)	0/23 (0.0%)	OR ∞ (95% CI 0.22–∞)	0.210	0.456
Multiple psychological/psychiatric categories (≥2)	4/20 (20.0%)	2/23 (8.7%)	OR 2.57 (95% CI 0.32–31.74)	0.393	0.601
Broad seizure semiology (harmonized domains)					
Positive motor phenomena	15/20 (75.0%)	16/23 (69.6%)	OR 1.30 (95% CI 0.28–6.45)	0.745	0.922
Negative motor phenomena	4/20 (20.0%)	2/23 (8.7%)	OR 2.57 (95% CI 0.32–31.74)	0.393	0.601
Impaired responsiveness/contact	12/20 (60.0%)	6/23 (26.1%)	OR 4.10 (95% CI 0.99–18.96)	0.033	0.144
Preserved awareness/responsiveness	7/20 (35.0%)	3/23 (13.0%)	OR 3.48 (95% CI 0.65–24.70)	0.148	0.385
Falls/collapse	3/20 (15.0%)	1/23 (4.3%)	OR 3.77 (95% CI 0.27–212.88)	0.323	0.601
Speech/language disturbance	4/20 (20.0%)	2/23 (8.7%)	OR 2.57 (95% CI 0.32-31.74)	0.393	0.601
Sensory/perceptual symptoms	3/20 (15.0%)	2/23 (8.7%)	OR 1.83 (95% CI 0.19–24.23)	0.650	0.846
Autonomic/visceral symptoms	1/20 (5.0%)	1/23 (4.3%)	OR 1.15 (95% CI 0.01–94.97)	1.000	1.000
Prolonged event ≥ 10 min	1/20 (5.0%)	1/23 (4.3%)	OR 1.15 (95% CI 0.01–94.97)	1.000	1.000
Neuroimaging					
Brain MRI performed	10/20 (50.0%)	18/22 (81.8%)	OR 0.23 (95% CI 0.04–1.06)	0.049	0.181
Abnormal brain MRI among imaged patients	0/10 (0.0%)	12/18 (66.7%)	OR 0.00 (95% CI 0.00–0.34)	**<0.001**	0.006
Treatment context					
Psychological/psychiatric treatment recorded	13/20 (65.0%)	3/23 (13.0%)	OR 11.52 (95% CI 2.28–82.26)	**<0.001**	0.005
ASM use at NES diagnosis/epilepsy assessment	8/20 (40.0%)	23/23 (100.0%)	OR 0.00 (95% CI 0.00–0.17)	**<0.001**	<0.001

NES-only denotes NES without comorbid epilepsy; epilepsy-only controls had epilepsy and no NES. Given the exploratory nature of the analysis, Benjamini–Hochberg false-discovery-rate (BH-FDR) adjustment was applied across the 26 comparisons in this table for which an inferential contrast was estimable. Binary comparisons used two-sided Fisher exact tests with conditional exact ORs; age used the Mann-Whitney U test. The MRI-abnormality analysis is conditional on having undergone MRI; MRI-performance status was available for 22/23 epilepsy controls. Treatment variables are shown for clinical context but should not be interpreted as diagnostic markers because timing and indication are not fully equivalent between groups. Recorded neurological comorbidity is a heterogeneous contextual construct. Bold identifies BH-FDR-adjusted *p* < 0.05; treatment-context rows remain interpretively secondary despite statistical significance.

Two exploratory sensitivity analyses were performed. First, the principal NES-only versus epilepsy-only findings were re-examined after restricting the NES-only group to patients with database-recorded video-EEG confirmation (*n* = 15). Second, the association between documented psychological/psychiatric comorbidity and diagnostic group was examined with Firth penalized logistic regression adjusted for age at first neurological visit and sex. This model was used as a small-sample sensitivity analysis rather than as a diagnostic prediction model.

Statistical analyses were performed in Python (version 3.13.5). Missing values were handled by complete case analysis for each variable, and no data imputation was performed.

## Results

Compared with epilepsy-only patients, NES-only patients had a markedly higher frequency of documented psychological/psychiatric comorbidity (90.0% vs. 13.0%; exact OR 50.40, 95% CI 7.29–670.28; BH-FDR *p* < 0.001), particularly anxiety (45.0% vs. 4.3%; exact OR 16.82, 95% CI 1.93–823.67; BH-FDR *p* = 0.014); although the literature reports a high prevalence of previous trauma and PTSD amongst patients with NES (Nicholson et al., 2026), we did not find a high prevalence of PTSD in our cohort. In particular, with reference to Table 1, we have classified these patients under the category “Other psychological/psychiatric comorbidity,” which includes three patients (7.9%) with disorders other than those explicitly listed. Impaired responsiveness/contact was numerically more frequent in NES-only (60.0% vs. 26.1%; raw *p* = 0.033), but this difference did not remain significant after BH-FDR correction (adjusted *p* = 0.144). Other broad harmonized seizure-semiology domains likewise did not differ significantly after multiplicity correction. Among imaged patients, MRI findings coded as abnormal were present in 0/10 NES-only patients and 12/18 epilepsy-only patients (exact OR 0.00, 95% CI 0.00–0.34; BH-FDR *p* = 0.006). MRI had, however, been performed in only 50.0% of NES-only patients versus 81.8% of epilepsy controls with available MRI status, and the MRI-performance comparison did not remain significant after correction (BH-FDR *p* = 0.181). The abnormal-MRI comparison is therefore conditional on an imaged subset and vulnerable to differential imaging ascertainment; structural findings may also contribute to establishing an epilepsy diagnosis. Treatment-context variables differed as well: psychological/psychiatric treatment was recorded in 65.0% versus 13.0% (BH-FDR *p* = 0.005), and ASM use at the recorded assessment point in 40.0% versus 100.0% (BH-FDR *p* < 0.001). These treatment comparisons are retained for clinical context but are not interpreted as diagnostic predictors because timing, indication, and care pathways were not fully harmonized between groups (Table 1).

The sensitivity analyses yielded a similar psychiatric-comorbidity pattern. Restricting NES-only cases to the 15 patients with database-recorded video-EEG confirmation, documented psychological/psychiatric comorbidity remained more frequent than in epilepsy-only controls (13/15 [86.7%] vs. 3/23 [13.0%]; exact OR 36.58, 95% CI 5.08–497.31; Fisher *p* < 0.001). In a Firth penalized logistic-regression sensitivity analysis adjusted for age at first neurological visit and sex, documented psychological/psychiatric comorbidity remained associated with NES-only group membership (adjusted OR 29.94; profile penalized-likelihood 95% CI 6.50–196.83; penalized likelihood-ratio *p* < 0.001). This model was used for confounding-adjusted sensitivity analysis rather than diagnostic prediction.

Among the 38 patients with NES, 31/38 (81.6%) were female and median age at NES diagnosis was 31.0 years [IQR 22.5–51.5]. Comorbid epilepsy was present in 18/38 (47.4%). Documented psychological/psychiatric comorbidity was present in 36/38 (94.7%), most commonly anxiety (19/38, 50.0%) and depression (11/38, 28.9%). Positive motor phenomena were the most frequently documented semiological domain (28/38, 73.7%), and inconsistency/incongruity on neurological examination was recorded in 30/35 evaluable patients (85.7%). Database-recorded video-EEG confirmation was available in 28/38 (73.7%), while epileptiform EEG activity was recorded in 5/38 (13.2%) (among patients with NES, isolated theta activity was observed in six cases and beta activity in one case; in patients with comorbid NES and epilepsy, epileptiform abnormalities were detected in five cases during EEG/video-EEG monitoring). Brain MRI was coded as abnormal in 6/38 (15.8%), negative in 15/38 (39.5%), and not performed in 17/38 (44.7%). At NES diagnosis, 24/38 (63.2%) were receiving an ASM; psychological/psychiatric treatment status was available for 37 patients and was positive in 21/37 (56.8%). Among the 20 patients with formal post-treatment follow-up, 8 (40.0%) were documented as improved, 11 (55.0%) unchanged, and 1 (5.0%) worsened (Table 2).

**TABLE 2:** Detailed descriptive phenotype of the 38 non-epileptic seizures (NES) patients.

Characteristic	Result
Demographics and comorbidity	
Female sex	31/38 (81.6%)
Age at NES diagnosis, years	31.0 [22.5–51.5]; range 14–64
Age at first neurological visit, years	30.0 [19.5–48.5]
Comorbid epilepsy	18/38 (47.4%)
Recorded neurological comorbidity	15/38 (39.5%)
Any documented psychological/psychiatric comorbidity	36/38 (94.7%)
Anxiety	19/38 (50.0%)
Depression	11/38 (28.9%)
Panic attacks	4/38 (10.5%)
Bipolar disorder	1/38 (2.6%)
Eating disorder	2/38 (5.3%)
Other psychological/psychiatric comorbidity	3/38 (7.9%)
Personality disorder	2/38 (5.3%)
Psychosis	4/38 (10.5%)
Multiple psychological/psychiatric categories (≥2)	9/38 (23.7%)
NES burden and core semiology	
NES frequency/month	3.0 [1.0–20.0]; range 0–300 (*n* = 37)
Positive motor phenomena	28/38 (73.7%)
Impaired responsiveness/contact	19/38 (50.0%)
Preserved awareness/responsiveness	18/38 (47.4%)
Negative motor phenomena (ictal only)	5/38 (13.2%)
Falls/collapse	5/38 (13.2%)
Speech/language disturbance	5/38 (13.2%)
Sensory/perceptual symptoms	5/38 (13.2%)
Dissociative/derealization phenomena	4/38 (10.5%)
Autonomic/visceral symptoms	2/38 (5.3%)
Positive functional ictal features and context	
Ictal variability/fluctuation/incongruity	4/38 (10.5%)
Ictal distractibility/modifiability	6/38 (15.8%)
Asynchronous/asymmetric motor pattern	2/38 (5.3%)
Ictal eye closure/opposition to opening	4/38 (10.5%)
Rapid recovery/no postictal confusion	3/38 (7.9%)
Explicit stress/emotional association	7/38 (18.4%)
Prolonged event ≥ 10 min	2/38 (5.3%)
Investigations, treatment and follow-up	
Database-recorded video-EEG confirmation	28/38 (73.7%)
Inconsistency/incongruity at neurological examination	30/35 (85.7%)
Epileptiform EEG activity	5/38 (13.2%)
Brain MRI: negative/abnormal/not performed	15 (39.5%)/6 (15.8%)/17 (44.7%)
ASM use at NES diagnosis	24/38 (63.2%)
Psychological/psychiatric treatment recorded	21/37 (56.8%)
Formal post-treatment follow-up available	20/38 (52.6%)
Outcome among formally followed patients	Improved 8/20 (40.0%); unchanged 11/20 (55.0%); worsened 1/20 (5.0%)

After correction for the 12 secondary comparisons reported in Table 3, no between-subgroup difference remained statistically significant between NES-only and NES with comorbid epilepsy for the measured demographic, NES-frequency, psychiatric-comorbidity, or broad semiological variables. Abnormal brain MRI findings among imaged patients (0/10 vs. 6/11; raw *p* = 0.012) and epileptiform EEG activity (0/20 vs. 5/18; raw *p* = 0.017) were concentrated in the comorbid-epilepsy subgroup, but neither remained statistically significant after BH-FDR correction (both adjusted *p* = 0.102) (Table 3). These instrumental findings are clinically coherent with the epilepsy component and may partly reflect incorporation of EEG or imaging evidence into the diagnosis of epilepsy; they should not be interpreted as NES biomarkers.

**TABLE 3:** Non-epileptic seizures (NES)-only versus NES with comorbid epilepsy: secondary exploratory comparison.

Variable	NES-only (*n* = 20)	NES + epilepsy (*n* = 18)	Effect estimate	*P*-value	BH-FDR *p*
Age at NES diagnosis, years	31.0 [24.8–49.8]	33.0 [22.5–51.5]	Rank-biserial *r* = −0.05	0.804	1.000
NES frequency/month	3.5 [0.8–21.2]	3.0 [1.0–20.0]	Rank-biserial *r* = 0.00	1.000	1.000
Female sex	17/20 (85.0%)	14/18 (77.8%)	OR 1.60 (95% CI 0.23–12.81)	0.687	1.000
Any documented psychological/psychiatric comorbidity	18/20 (90.0%)	18/18 (100.0%)	OR 0.00 (95% CI 0.00–5.88)	0.488	1.000
Positive motor phenomena	15/20 (75.0%)	13/18 (72.2%)	OR 1.15 (95% CI 0.21–6.28)	1.000	1.000
Impaired responsiveness/contact	12/20 (60.0%)	7/18 (38.9%)	OR 2.30 (95% CI 0.54–10.59)	0.330	0.990
Preserved awareness/responsiveness	7/20 (35.0%)	11/18 (61.1%)	OR 0.35 (95% CI 0.07–1.53)	0.193	0.771
Inconsistency/incongruity at neurological examination	16/19 (84.2%)	14/16 (87.5%)	OR 0.77 (95% CI 0.06–7.75)	1.000	1.000
Database-recorded video-EEG confirmation	15/20 (75.0%)	13/18 (72.2%)	OR 1.15 (95% CI 0.21–6.28)	1.000	1.000
Brain MRI performed	10/20 (50.0%)	11/18 (61.1%)	OR 0.64 (95% CI 0.14–2.76)	0.532	1.000
Abnormal MRI among imaged patients	0/10 (0.0%)	6/11 (54.5%)	OR 0.00 (95% CI 0.00–0.66)	0.012	0.102
Epileptiform EEG activity	0/20 (0.0%)	5/18 (27.8%)	OR 0.00 (95% CI 0.00–0.86)	0.017	0.102

### Exploratory three-group analysis

The three-group extension considered NES-only (*n* = 20), NES + epilepsy (*n* = 18), and epilepsy-only (*n* = 23). It should be regarded as an exploratory re-expression of the same cohort rather than as independent replication of the primary findings (Figure 1).

**FIGURE 1:**
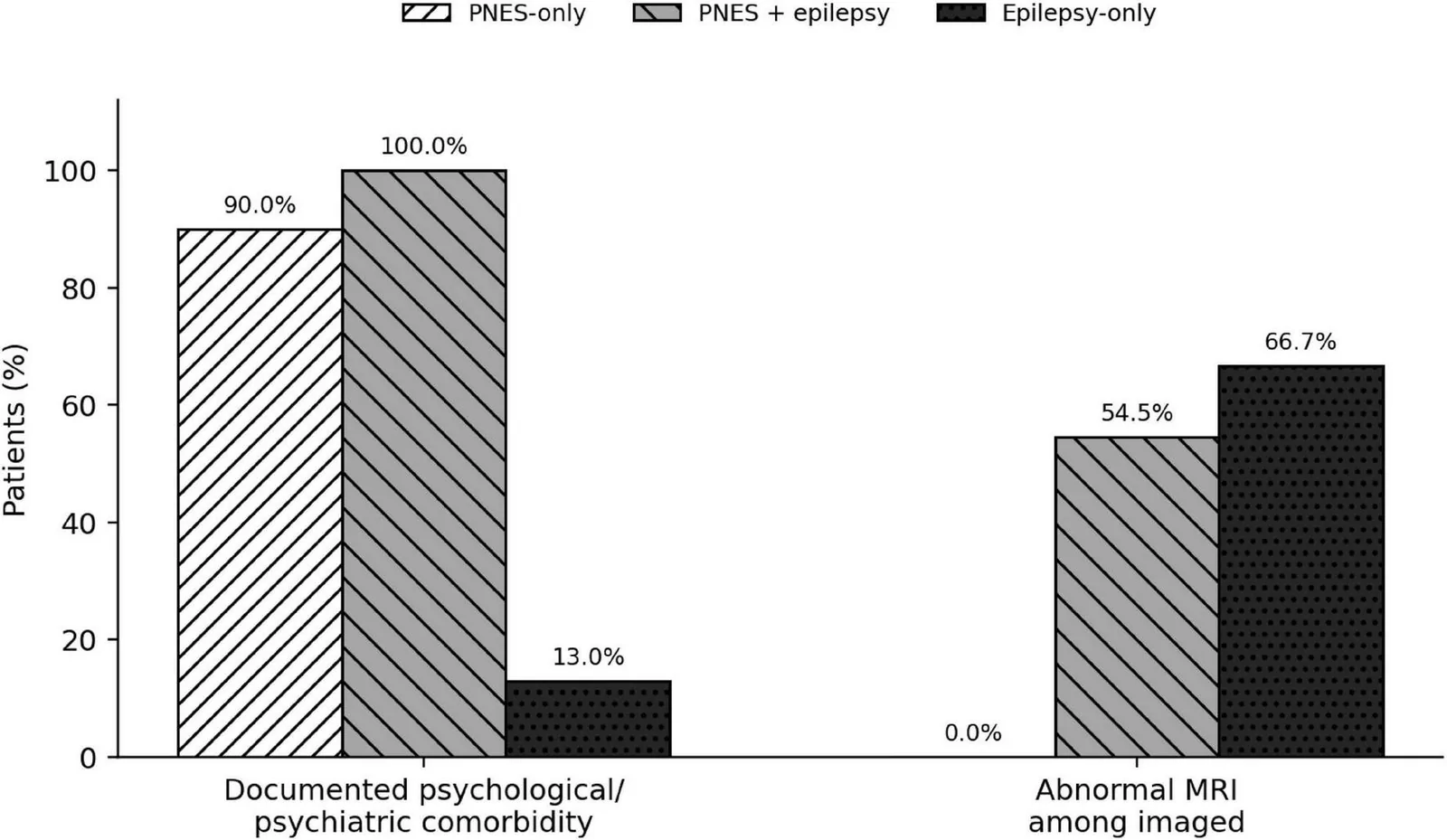
Exploratory three-group patterns for documented psychological/psychiatric comorbidity and abnormal MRI among imaged patients. Psychological/psychiatric comorbidity: 90.0%, 100.0%, and 13.0%, respectively; exact Fisher-Freeman-Halton *p* < 0.001, Cramer’s V = 0.83. Abnormal MRI among imaged patients: 0.0%, 54.5%, and 66.7%; exact *p* = 0.0014, Cramer’s V = 0.55. These selected exploratory patterns are a re-expression of the same cohort and do not constitute independent validation of the primary comparison.

In the three-group analysis, documented psychiatric comorbidity was more frequent in both groups containing NES than in epilepsy-only controls, whereas abnormal MRI findings were concentrated in the two groups containing epilepsy. This exploratory analysis re-expresses the same cohort and should not be interpreted as evidence of causation or of a mechanistic distinction based on MRI.

## Discussion

This retrospective comparative study characterizes a clinically heterogeneous NES cohort and examines differences across NES-only, NES with comorbid epilepsy, and epilepsy-only groups. The modest, single-center sample and retrospective ascertainment limit precision, so the analyses are intended primarily to describe clinically relevant patterns rather than to derive diagnostic prediction rules.

Female predominance was observed in the NES cohort (31/38, 81.6%), consistent with the demographic pattern frequently reported in NES populations. Because the sample was relatively small and derived from a single referral setting, the observed proportion should be viewed as a characteristic of this cohort rather than as a population estimate.

Psychological/psychiatric comorbidity was frequently documented in the NES cohort, with anxiety and depression the most common categories. The contrast between NES-only and epilepsy-only remained large in the video-EEG-restricted analysis and after age/sex adjustment with Firth penalized logistic regression. This pattern is consistent with the established association between NES and psychiatric morbidity (Brown and Reuber, 2016; Gasparini et al., 2019; Lanzillotti et al., 2021; Walsh et al., 2018), but the magnitude requires caution: psychiatric history may have been elicited or recorded more intensively during NES assessment than during routine epilepsy care. Accordingly, the 90.0% versus 13.0% difference describes documented comorbidity in this dataset and should not be interpreted as an unbiased population-prevalence estimate or as evidence of psychiatric causation; the prevalence we found of 13.0% is well in line with results from previous studies (Kwon et al., 2025).

After multiplicity correction, none of the measured core NES features differed significantly between patients with and without comorbid epilepsy. Impaired responsiveness was numerically more frequent in NES-only, whereas preserved responsiveness was numerically more frequent in the comorbid-epilepsy subgroup, but the confidence intervals were wide. With subgroup sizes of 20 and 18 patients, these findings do not demonstrate equivalence and cannot exclude clinically meaningful differences in NES phenotype.

The semiological findings illustrate the heterogeneity of NES. Positive motor phenomena were the most frequently documented domain, whereas specific positive functional ictal features such as ictal eye closure, asynchronous movements, distractibility, and fluctuating/incongruent ictal behavior were documented in smaller subsets. This may reflect both true phenotypic heterogeneity and the limitations of retrospective narrative documentation. Inconsistency/incongruity on neurological examination was recorded in 85.7% of evaluable NES patients, demonstrating that positive functional neurological signs were common in this cohort; however, their sensitivity, specificity, and independent diagnostic contribution to the seizure diagnosis were not assessed. Previous literature has identified several ictal signs as potentially useful discriminators, but no single feature should be used in isolation and interpretation must remain contextual, with video-EEG used when clinically appropriate (Asadi-Pooya and Homayoun, 2020; Kerr, 2023; Labudda et al., 2020; von Elm et al., 2007).

Within the NES cohort, abnormal MRI findings and epileptiform EEG activity were observed only in patients with comorbid epilepsy, although the subgroup differences did not remain significant after the single-family BH-FDR correction. In the primary comparison, abnormal MRI findings were also more frequent among imaged epilepsy-only patients than imaged NES-only patients. These observations are clinically coherent with the epilepsy component of the sample and with previous reports of structural abnormalities in NES cohorts with comorbid epilepsy (Kerr, 2023; von Elm et al., 2007; Whitehead et al., 2017), but they are partly susceptible to incorporation/circularity because structural or epileptiform abnormalities may contribute to the clinical diagnosis of epilepsy. Differential MRI ascertainment further limits direct prevalence interpretation. These findings should therefore characterize the sample rather than be presented as newly identified biomarkers.

Database-recorded video-EEG confirmation was available in approximately three-quarters of NES patients. The study did not evaluate the diagnostic accuracy or clinical impact of video-EEG, so this proportion should not be interpreted as a measure of diagnostic performance. Restriction to NES-only patients with recorded video-EEG confirmation reproduced the main psychiatric-comorbidity association. Within the NES cohort, epileptiform EEG activity was recorded only among patients with comorbid epilepsy, underscoring the need to distinguish coexisting epileptic and non-epileptic event types rather than assume diagnostic exclusivity.

Treatment and follow-up data were descriptive. More than half of the NES cohort had documented psychological/psychiatric treatment, and many were receiving ASMs at the time of NES diagnosis; however, indication and timing were heterogeneous, particularly in patients with comorbid epilepsy. Among the 20 patients with formal follow-up, 40.0% were documented as improved, 55.0% unchanged, and 5.0% worsened. Because treatment exposure, follow-up duration, and the post-treatment observation window were not standardized, these data cannot support causal conclusions regarding treatment effectiveness. Prospective studies with predefined interventions and follow-up intervals are needed to evaluate outcome determinants (Asadi-Pooya and Homayoun, 2020; Gasparini et al., 2019; Goldstein et al., 2010; Kerr, 2023).

## Conclusion

In this cohort, NES were clinically heterogeneous and frequently coexisted with epilepsy. No broad seizure-semiology domain differed significantly between NES-only and epilepsy-only after correction for multiple comparisons. Documented psychological/psychiatric comorbidity, particularly anxiety, was substantially more frequent in NES-only and remained associated with NES status in sensitivity analyses, although differential psychiatric ascertainment is an important alternative explanation. We found no statistically significant evidence that comorbid epilepsy changed the measured NES phenotype, but the modest subgroup sizes preclude claims of equivalence. Abnormal MRI findings were concentrated in groups containing epilepsy, while within the NES cohort epileptiform EEG activity was recorded only in patients with comorbid epilepsy. These instrumental findings are best interpreted as descriptive clinical characteristics rather than NES biomarkers. Future studies should stratify explicitly for comorbid epilepsy and use more standardized, independently coded semiological and psychiatric assessments.

## Limitations

Several limitations should be considered. First, the sample was modest, particularly after stratification into NES-only and NES with comorbid epilepsy, resulting in wide confidence intervals and limited power for rare features; non-significant comparisons therefore should not be interpreted as evidence of equivalence. Second, the retrospective single-center design and the different clinical pathways through which NES and epilepsy patients entered the databases may have introduced selection and referral bias. Third, psychiatric comorbidity was derived from clinical documentation rather than a uniform standardized research assessment, creating potential differential ascertainment between groups. Fourth, semiology was reconstructed from clinical narratives, documentation depth differed between groups, and absence of a recorded feature could not be assumed to indicate clinical absence; the analytic coding is an operational categorization rather than a validated diagnostic scale. Fifth, database-recorded video-EEG confirmation was available in 28/38 NES patients rather than the entire cohort, so diagnostic certainty was not uniform; a restricted sensitivity analysis was therefore reported. Sixth, MRI analyses were conditional on undergoing imaging, which occurred with different frequency across groups, and MRI/EEG abnormalities may have contributed to epilepsy ascertainment. Seventh, treatment variables were not fully harmonized in timing or indication, and post-treatment exposure and follow-up duration were not sufficiently standardized for causal effectiveness inference. Moreover, multiple clinical variables were examined in a small dataset; BH-FDR correction reduced false-positive risk, but subgroup and three-group analyses remain exploratory and clinically meaningful differences may have gone undetected. Finally, because patients with other neurologic co-morbidities were excluded this limits generalizability due to the fact that there is often prevalence of co-morbid medical and other neurologic conditions in patients with NES.

## Data Availability

The raw data supporting the conclusions of this article will be made available by the authors, without undue reservation.

## References

[B1] Anzellotti F. Dono F. Evangelista G. Di Pietro M. Carrarini C. Russo M. et al. (2020). Psychogenic non-epileptic seizures and pseudo-refractory epilepsy, a management challenge. *Front. Neurol.* 11:461. 10.3389/fneur.2020.00461 32582005 PMC7280483

[B2] Asadi-Pooya A. A. (2023). Incidence and prevalence of psychogenic nonepileptic seizures (functional seizures): A systematic review and an analytical study. *Int. J. Neurosci.* 133, 598–603. 10.1080/00207454.2021.1942870 34126844

[B3] Asadi-Pooya A. A. Homayoun M. (2020). Structural brain abnormalities in patients with psychogenic nonepileptic seizures. *Neurol. Sci.* 41, 555–559. 10.1007/s10072-019-04108-7 31713756

[B4] Brown R. J. Reuber M. (2016). Psychological and psychiatric aspects of Psychogenic Non-Epileptic Seizures (PNES): A systematic review. *Clin. Psychol. Rev.* 45, 157–182. 10.1016/j.cpr.2016.01.003 27084446

[B5] Gasparini S. Beghi E. Ferlazzo E. Beghi M. Belcastro V. Biermann K. P. et al. (2019). Management of psychogenic non-epileptic seizures: A multidisciplinary approach. *Eur. J. Neurol.* 26, 205–e15. 10.1111/ene.13818 30300463

[B6] Goldstein L. H. Chalder T. Chigwedere C. Khondoker M. R. Moriarty J. Toone B. K. et al. (2010). Cognitive-behavioral therapy for psychogenic nonepileptic seizures: A pilot RCT. *Neurology* 74, 1986–1994. 10.1212/WNL.0b013e3181e39658 20548043 PMC2905892

[B7] Hingray C. Popkirov S. Kozlowska K. Pretorius C. Sarudiansky M. El-Hage W. et al. (2025). Functional/dissociative seizures: Proposal for a new diagnostic label and definition by the ILAE task force. *Epilepsia* 66, 4162–4182. 10.1111/epi.18574 40884444 PMC12661283

[B8] Jungilligens J. Michaelis R. Popkirov S. (2021). Misdiagnosis of prolonged psychogenic non-epileptic seizures as status epilepticus: Epidemiology and associated risks. *J. Neurol. Neurosurg. Psychiatry* 92, 1341–1345. 10.1136/jnnp-2021-326443 34362852 PMC8606439

[B9] Kanemoto K. LaFrance W. C. Duncan R. Gigineishvili D. Park S. P. Tadokoro Y. et al. (2017). PNES around the world: Where we are now and how we can close the diagnosis and treatment gaps-an ILAE PNES Task Force report. *Epilepsia Open* 2, 307–316. 10.1002/epi4.12060 29588959 PMC5862115

[B10] Kerr W. T. (2023). Using verbally-reported and video-observed semiology to identify functional seizures. *Neurol. Clin.* 41, 605–617. 10.1016/j.ncl.2023.02.003 37775193

[B11] Kutlubaev M. A. Xu Y. Hackett M. L. Stone J. (2018). Dual diagnosis of epilepsy and psychogenic nonepileptic seizures: Systematic review and meta-analysis of frequency, correlates, and outcomes. *Epilepsy Behav.* 89, 70–78. 10.1016/j.yebeh.2018.10.010 30384103

[B12] Kwon C. S. Rafati A. Ottman R. Christensen J. Kanner A. M. Jetté N. et al. (2025). Psychiatric comorbidities in persons with epilepsy compared with persons without epilepsy: A systematic review and meta-analysis. *JAMA Neurol.* 82, 72–84. 10.1001/jamaneurol.2024.3976 39585664 PMC11589854

[B13] Labudda K. Frauenheim M. Miller I. Schrecke M. Brandt C. Bien C. G. (2020). Outcome of CBT-based multimodal psychotherapy in patients with psychogenic nonepileptic seizures: A prospective naturalistic study. *Epilepsy Behav.* 106:107029. 10.1016/j.yebeh.2020.107029 32213454

[B14] LaFrance W. C. Baker G. A. Duncan R. Goldstein L. H. Reuber M. (2013). Minimum requirements for the diagnosis of psychogenic nonepileptic seizures: A staged approach: A report from the international league against epilepsy nonepileptic seizures task force. *Epilepsia* 54, 2005–2018. 10.1111/epi.12356 24111933

[B15] Lanzillotti A. I. Sarudiansky M. Lombardi N. R. Korman G. P. D Alessio L. (2021). Updated review on the diagnosis and primary management of psychogenic nonepileptic seizure disorders. *Neuropsychiatr. Dis. Treat.* 17, 1825–1838. 10.2147/NDT.S286710 34113112 PMC8187153

[B16] Mostacci B. Bisulli F. Alvisi L. Licchetta L. Baruzzi A. Tinuper P. (2011). Ictal characteristics of psychogenic nonepileptic seizures: What we have learned from video/EEG recordings–a literature review. *Epilepsy Behav.* 22, 144–153. 10.1016/j.yebeh.2011.07.003 21856237

[B17] Nicholson D. Gaskell C. Rawlings G. H. Gray C. Xue Q. Reuber M. et al. (2026). The prevalence of post-traumatic stress disorder in functional / dissociative seizures: A systematic review and meta-analysis. *Seizure* 141, 223–232. 10.1016/j.seizure.2026.07.027 42541980

[B18] Reuber M. Fernández G. Bauer J. Helmstaedter C. Elger C. E. (2002). Diagnostic delay in psychogenic nonepileptic seizures. *Neurology* 58, 493–495. 10.1212/wnl.58.3.493 11839862

[B19] Viarasilpa T. Panyavachiraporn N. Osman G. Akioyamen N. O. Wasade V. S. Barkley G. et al. (2019). Intubation for psychogenic non-epileptic attacks: Frequency, risk factors, and impact on outcome. *Seizure* 76, 17–21. 10.1016/j.seizure.2019.12.025 31958705

[B20] von Elm E. Altman D. G. Egger M. Pocock S. J. Gøtzsche P. C. Vandenbroucke J. P. (2007). The Strengthening the Reporting of Observational Studies in Epidemiology (STROBE) statement: Guidelines for reporting observational studies. *Lancet* 370, 1453–1457. 10.1016/S0140-6736(07)61602-X 18064739

[B21] Walsh S. Levita L. Reuber M. (2018). Comorbid depression and associated factors in PNES versus epilepsy: Systematic review and meta-analysis. *Seizure* 60, 44–56. 10.1016/j.seizure.2018.05.014 29906707

[B22] Whitehead K. Kane N. Wardrope A. Kandler R. Reuber M. (2017). Proposal for best practice in the use of video-EEG when psychogenic non-epileptic seizures are a possible diagnosis. *Clin. Neurophysiol. Pract.* 2, 130–139. 10.1016/j.cnp.2017.06.002 30214985 PMC6123876

